# Prospective assessment of health-related quality of life in early phase oncology clinical trials: PEARLER

**DOI:** 10.1093/jncics/pkaf108

**Published:** 2025-11-10

**Authors:** Udit Nindra, Joanne Tang, Jun Hee Hong, Joseph Descallar, Martin Hong, Andrew Killen, Priyadarshini Dubey, Jeneen Attaullah, Grace Scott, Adam Cooper, Kate Wilkinson, Abhijit Pal, Christina Teng, Aflah Roohullah, Joe Wei, Weng Ng, Charlotte Lemech, Wei Chua

**Affiliations:** Department of Medical Oncology, Wollongong Hospital, Wollongong, Australia; Department of Medicine, University of Wollongong, Wollongong, Australia; Department of Medical Oncology, Liverpool Hospital, Liverpool, Australia; Department of Medicine, Western Sydney University, Campbelltown, Australia; Department of Medical Oncology, Liverpool Hospital, Liverpool, Australia; Department of Medical Oncology, Liverpool Hospital, Liverpool, Australia; Department of Medicine, Western Sydney University, Campbelltown, Australia; Ingham Institute for Applied Medical Research, Liverpool, NSW, Australia; South Western Sydney Clinical Campuses, School of Clinical Medicine, UNSW Sydney, NSW, Australia; Department of Medical Oncology, Liverpool Hospital, Liverpool, Australia; Department of Medicine, Western Sydney University, Campbelltown, Australia; Scientia Clinical Research, Randwick, Australia; Ingham Institute for Applied Medical Research, Liverpool, NSW, Australia; Ingham Institute for Applied Medical Research, Liverpool, NSW, Australia; Scientia Clinical Research, Randwick, Australia; Department of Medical Oncology, Liverpool Hospital, Liverpool, Australia; Department of Medicine, Western Sydney University, Campbelltown, Australia; Department of Medical Oncology, Liverpool Hospital, Liverpool, Australia; Department of Medicine, Western Sydney University, Campbelltown, Australia; Department of Medical Oncology, Liverpool Hospital, Liverpool, Australia; Department of Medicine, Western Sydney University, Campbelltown, Australia; Scientia Clinical Research, Randwick, Australia; Department of Medical Oncology, Prince of Wales Hospital, Randwick, Australia; Department of Medical Oncology, Liverpool Hospital, Liverpool, Australia; Department of Medical Oncology, Campbelltown Hospital, Campbelltown, Australia; Scientia Clinical Research, Randwick, Australia; Department of Medical Oncology, Prince of Wales Hospital, Randwick, Australia; Department of Medical Oncology, Liverpool Hospital, Liverpool, Australia; Department of Medicine, Western Sydney University, Campbelltown, Australia; Scientia Clinical Research, Randwick, Australia; Department of Medical Oncology, Prince of Wales Hospital, Randwick, Australia; Department of Medical Oncology, Liverpool Hospital, Liverpool, Australia; Department of Medicine, Western Sydney University, Campbelltown, Australia

## Abstract

**Introduction:**

Health-related quality of life (HRQoL) is not routine in early phase clinical trials (EP-CTs), which focus on dose-limiting toxicities and safety. However, for clinicians, understanding the impact of such trials on HRQoL is fundamental to consent patients, especially when the benefits on tumor response may be unknown.

**Aims and Methods:**

The PEARLER (Patient diversity And experience in eaRLy phase cancEr clinical tRials) study was conducted with a key aim of focusing on assessing HRQoL in participants undergoing EP-CTs using a multi-center prospective cohort setting. All participants completed a baseline demographic survey on Cycle 1 Day 1 with EORTC-QLQ-C30 on Day 1 of Cycles 1 through 6 or end of treatment (EoT).

**Results:**

Overall, 122 participants were recruited with median age 62. Median baseline Global Health Status (GHS) was 67 and remained unchanged throughout EP-CT (*P* = .188). GHS deterioration occurred in 29/122 (24%) while improvement occurred in 16/122 (13%). Median baseline Physical Function Score (PFS) was 87. PFS deterioration occurred in 30/122 (25%) while improvement occurred in 6/122 (5%). Baseline median CFS was 84. Cognitive Function Score (CFS) deterioration occurred in 25/122 (20%) while improvement occurred in 20/122 (16%). Baseline median Emotional Function Score (EFS) was 77. EFS deterioration occurred in 14/122 (11%) while improvement occurred in 14/122 (11%). Presence of liver metastases was a negative predictive marker for GHS, CFS, and EFS over time (*P* = .01, *P* < .01, and *P* < .01).

**Conclusion:**

PEARLER is the first prospective cohort study investigating change in HRQoL over time in patients undergoing EP-CTs. Reassuringly, almost three-quarters of participants who undertake EP-CTs either sustain or improve their GHS or PFS. Presence of liver metastases appears to be a negative predictive marker of HRQoL.

## Introduction

Early phase clinical trials (EP-CTs) can provide patients with further therapeutic options once standard of care treatments are exhausted. These studies provide hope of additional tumor response and over the past 20 years, response rates in EP-CTs have been improving.^1^ While these studies have a key focus on establishing appropriate dose levels as well as detecting early signals in tumor response, and toxicities of concern, they typically do not focus on patient-reported outcomes to assess health-related quality of life (HRQoL). In fact, a recent systematic review demonstrated that across 1333 phase 1 studies only 15 (1.1%) had HRQoL as an endpoint.^2^ As a result, there remains significant apprehension for patients who enroll into these studies as to the value these studies can provide. The hope of cancer control and implied survival benefits should be balanced with growing concern regarding the demands these studies have on patients^3^ with life-limiting diagnoses, and if participants’ HRQoL is negatively impacted by trial enrolment.^2^

In addition to discussing the impact of EP-CTs on participants’ HRQoL at the time of enrolment, HRQoL assessments using patient-reported outcome measurements (PROMs) can also be used by researchers to guide maximum tolerated doses in studies. An example of this correlation was demonstrated by Anota et al. wherein the recommended phase 2 dose of idarubicin was correlated with patient-reported HRQoL and demonstrated consistent trends suggesting poorer outcomes with either insufficient or excess dosing.^4^ In order to further explore this, an exploratory survey of key experts in clinical drug development was conducted regarding the utility of PROM-based HRQoL in EP-CTs.^5^ Although the majority of respondents noted that PROMs help determine additional toxicity data that could guide endpoints in EP-CTs, barriers regarding additional workload for clinical staff and lack of understanding regarding PROM analysis remain. As such, although increasing research has been done to assess the role of patient-reported HRQoL assessments in EP-CTs, their routine clinical implementation is lacking.

To date, there has been no prospective data collection regarding HRQoL across EP-CTs in general, nor assessment of the feasibility of doing such research. This void in the research landscape was the inspiration for the PEARLER (Patient diversity And experience in eaRLy phase cancEr clinical tRials) study, the key aim of which was to assess HRQoL outcome measures in a prospective standardized manner.

## Methods

The PEARLER study was a multi-center, prospective, cohort study involving 2 major EP-CT centers in Sydney, Australia. All participants who were consented to an EP-CT at either center were invited to participate. For the purpose of this study, EP-CTs were defined as phase I or phase Ib oncology trials, including both dose-escalation and dose-expansion cohorts. Patients enrolled in phase II or later-phase studies were excluded. Recruitment was consecutive: all patients who consented to participate in a phase I or phase Ib trial at the 2 participating centers during the study period were approached and invited to participate in PEARLER. No additional exclusion criteria were applied beyond the eligibility criteria of the individual EP-CTs. Of 125 patients screened for EP-CT participation during the study period, 122 of these consented to PEARLER. All participants completed a baseline demographic survey on Cycle 1 Day 1 (Figure S1). Demographic data collected included patient cultural and linguistic diversity (CALD) status, sexual orientation, socioeconomic status, and regional diversity. In addition to this baseline survey, participants completed the EORTC-QLQ-C30 (Figure S2) HRQoL survey on Day 1 of Cycles 1 through 6. Cycle 6 was chosen as the final cycle as prior research in our units suggested that less than 10% of participants in EP-CTs continued treatment beyond Cycle 6. The project was approved by the Sydney South West Local Health District Human Research Ethics Committee (2023/ETH00786).

The EORTC-QLQ-C30 scoring manual was used to calculate composite scores for Global Health Status (GHS), Physical Function Score (PFS), Emotional Function Score (EFS), and Cognitive Function Score (CFS). A decrease in 10 points from baseline to end of EP-CT participation was defined as a significant change from baseline (CFB), denoting deterioration in the scores. Likewise, an increase in 10 points from baseline to end of EP-CT was denoted as improvement.^6^ Furthermore, prognostic scores such as the Royal Marsden Hospital Score (RMH) and MD Anderson Cancer Centre Score (MDACC) were calculated for each patient at baseline and correlated with changes in each of the scores.^7^^,^^8^ The RMH score was categorized into low (0-1) and high (2-3). The MDACC was also categorized into low (0-1) and high (2-4).

Multilevel models were used to analyze each of GHS, PFS, EFS, and CFS. Random linear models were specified and maximum likelihood was estimated with Sattherwaite degrees of freedom. For each model, cycle, ECOG category, ISRAD category, liver metastases, age category, EP-CT type, RMH category, and MDACC category were included in the initial multivariable models. Additionally, interactions between cycle and each of ECOG, ISRAD, liver metastases, age category, EP-CT, RMH category, and MDACC category were also included to investigate whether the effects of these variables differed over cycles within those categories. A backward stepwise regression was used to eliminate variables from the model with *P* < .05 considered as significant until the final model was reached. Cycle number remained in the models regardless of statistical significance. We did not adjust for trial-level heterogeneity (eg, differences in visit intensity or procedures) as sample sizes per trial were insufficient for stratified analyses. Missing HRQoL data were handled using a maximum likelihood approach within the mixed-effects modelling framework, which allows inclusion of participants with incomplete data across cycles under the assumption of missing at random. We did not perform imputation (eg, last observation carried forward), and participants contributed data for all completed cycles until attrition or end of treatment (EoT). Statistical analysis was conducted in R version 4.5 and SAS version 9.4.

## Results

### Patient demographics

**Figure 1.**
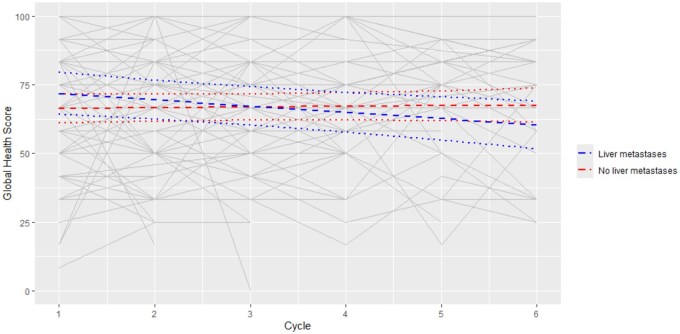
Global Health Status (GHS) during EP-CT participation stratified by liver metastases. Solid lines represent mean predicted GHS scores over cycles, with separate lines for patients with liver metastases and without liver metastases. Shaded bands/dashed lines represent 95% confidence intervals. Baseline median GHS for all participants was 67/100. This score is derived using the EORTC-QLQ-C30 Scoring Manual which converts the raw score out of 4 to a toxicity score out of 100. While there was no significant difference in the GHS between patients with and without liver metastases at any individual cycle, patients with liver metastases had a significant reduction of 2.29 in GHS per cycle (95% CI = −3.9 to −0.69, *P* = .0054).

Overall, 122 participants were recruited into the PEARLER study. Median age of participants was 62 years (25–83 years) with 63 (52%) participants identifying as female. Forty-seven (39%) participants were enrolled in immuno-oncology EP-CTs while the remainder participated in EP-CTs focused on targeted therapies. Twenty-one (17%) participants required a biopsy for enrolment into their EP-CTs. The majority (*n* = 82, 67%) of participants had a baseline European Cooperative Oncology Group (ECOG) performance score of 0 at the time of EP-CT enrolment. Complete baseline demographics are outlined in Table 1, and additional descriptive characteristics of the included trials are summarized in Table S1, including trial sponsorship, geographic location, mean number of participants per trial, and phase breakdown. A consort diagram is shown in Figure 1.

**Table 1.** Baseline participant demographics.

Demographic	No. (%)
Total participants	122
Age, median [range]	62 [25–83]
Self-reported gender	
Female	63 (52)
Male	59 (48)
Other	0 (0)
Self-reported sexuality	
Heterosexual	122 (100)
LGBTQIA+	0 (0)
ECOG PS	
0	82 (67)
1	40 (33)
Smoking status	
Never smoked	70 (57)
Current or ex-smoker	52 (43)
Cancer type	
Gynaecological	25 (20)
Lung	24 (20)
Upper gastrointestinal	14 (11)
Colorectal	14 (11)
Head and neck	12 (10)
Brain	9 (7)
Skin	6 (5)
Breast	4 (3)
Other	14 (11)
CALD status	
Culturally diverse	39 (32)
Linguistically diverse	32 (26)
Indigenous Australian	1 (1)
Country of birth	
Australia	87 (71)
Overseas	35 (29)
Socioeconomic status	
IRSAD 4-5	65 (53)
IRSAD ≤ 3	57 (47)
Living status	
Living with family	113 (93)
Living alone	9 (7)
Clinical trial phase	
Dose escalation	88 (72)
Dose expansion	34 (28)
Type of investigational product	
Targeted therapy	75 (61)
Immuno-oncology	47 (39)

Abbreviations: CALD = Culturally and Linguistically Diverse; IRSAD = Index of Relative Social Advantage and Disadvantage; ECOG PS = European Cooperative Oncology Group Performance Score; LGBTQIA+ = Lesbian, Gay, Bisexual, Transgender, Queer, Intersex, Asexual.

### Global Health Status

Among 122 participants, the median GHS at baseline was 67 and remained steady throughout EP-CT participation (*P* = .188). GHS deterioration, defined by a 10-point decrease from baseline to EoT, occurred in 29/122 (24%), while GHS improvement, defined by a 10-point increase from baseline to EoT, occurred in 16/122 (13%). The final multivariable model for GHS included cycle, liver metastases, and an interaction between liver metastases and cycle (*P* = .0145). While there was no significant difference in the GHS between patients with and without liver metastases at any individual cycle, patients with liver metastases had a significant reduction of 2.29 in GHS per cycle (95% CI = −3.9 to −0.69, *P* = .0054) (Figure 1). Age, CALD, ISRED EP-CT type, RMH, and MDACC were not associated with GHS.

### Physical Function Score

**Figure 2.**
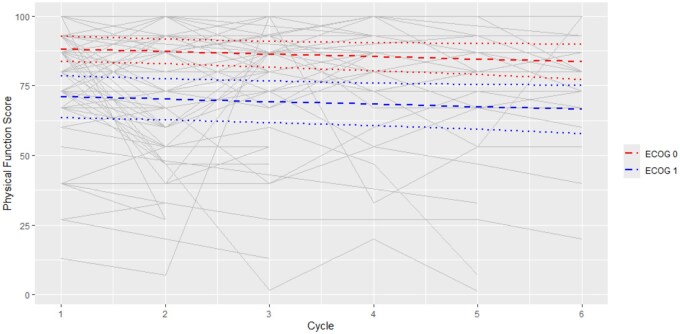
Baseline Physical Function score pre-Cycle 1 for all participants was 83/100. This score is derived using the EORTC-QLQ-C30 Scoring Manual which converts the raw score out of 4 to a toxicity score out of 100. While there was no evidence of change over cycles in PFS (Coefficient = −0.91, 95% CI = −2 to 0.19, *P* = .1051), there was on average a 17.1 higher PFS for patients with lower ECOG scores (95% CI = 8.54 to 25.74, *P* = .0001).

Among 122 participants, the median PFS at baseline was 83. PFS deterioration, defined by a 10-point decrease from baseline to EoT, occurred in 30/122 (25%), while GHS improvement, defined by a 10-point increase from baseline to EoT, occurred in 6/122 (5%). Changes in PFS over time are demonstrated in Figure 2. While there was no evidence of change over cycles in PFS (Coefficient = −0.91, 95% CI = −2 to 0.19, *P* = .1051), there was on average of 17.1 higher PFS for patients with lower ECOG scores (95% CI = 8.54 to 25.74, *P* = .0001). Age, CALD, ISRAD, liver metastases, EP-CT type, RMH, and MDACC were not significantly associated with PFS.

### Cognitive Function Score

**Figure 3.**
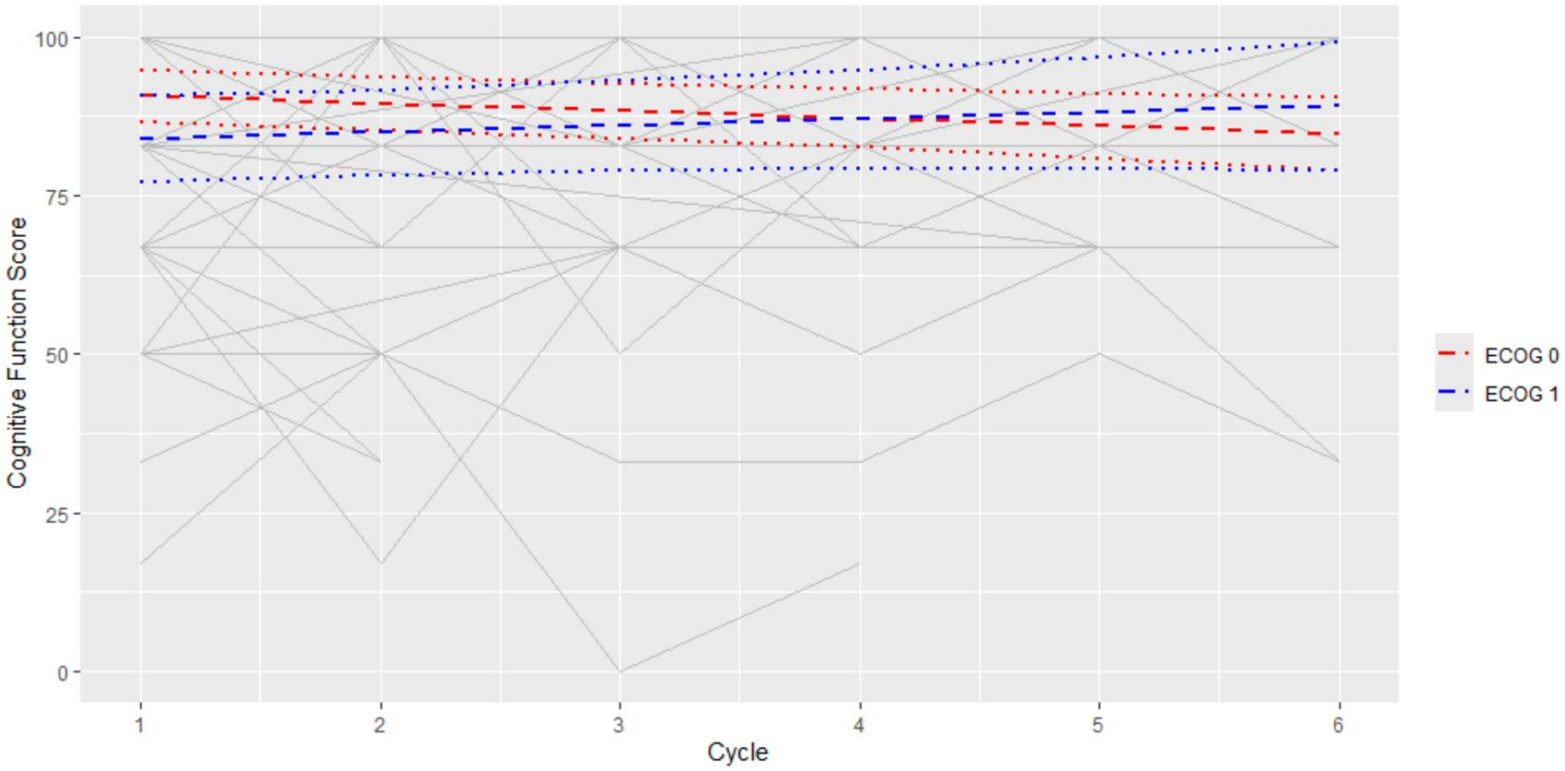
Among 122 participants, the median CFS at baseline was 84. This score is derived using the EORTC-QLQ-C30 Scoring Manual which converts the raw score out of 4 to a toxicity score out of 100. The individual grey lines are data points from the individual participants. A significant reduction in CFS was observed for patients in lower ECOG groups of −1.2 per cycle (95% CI = −2.1 to −0.2, *P* = .0165).

**Figure 4.**
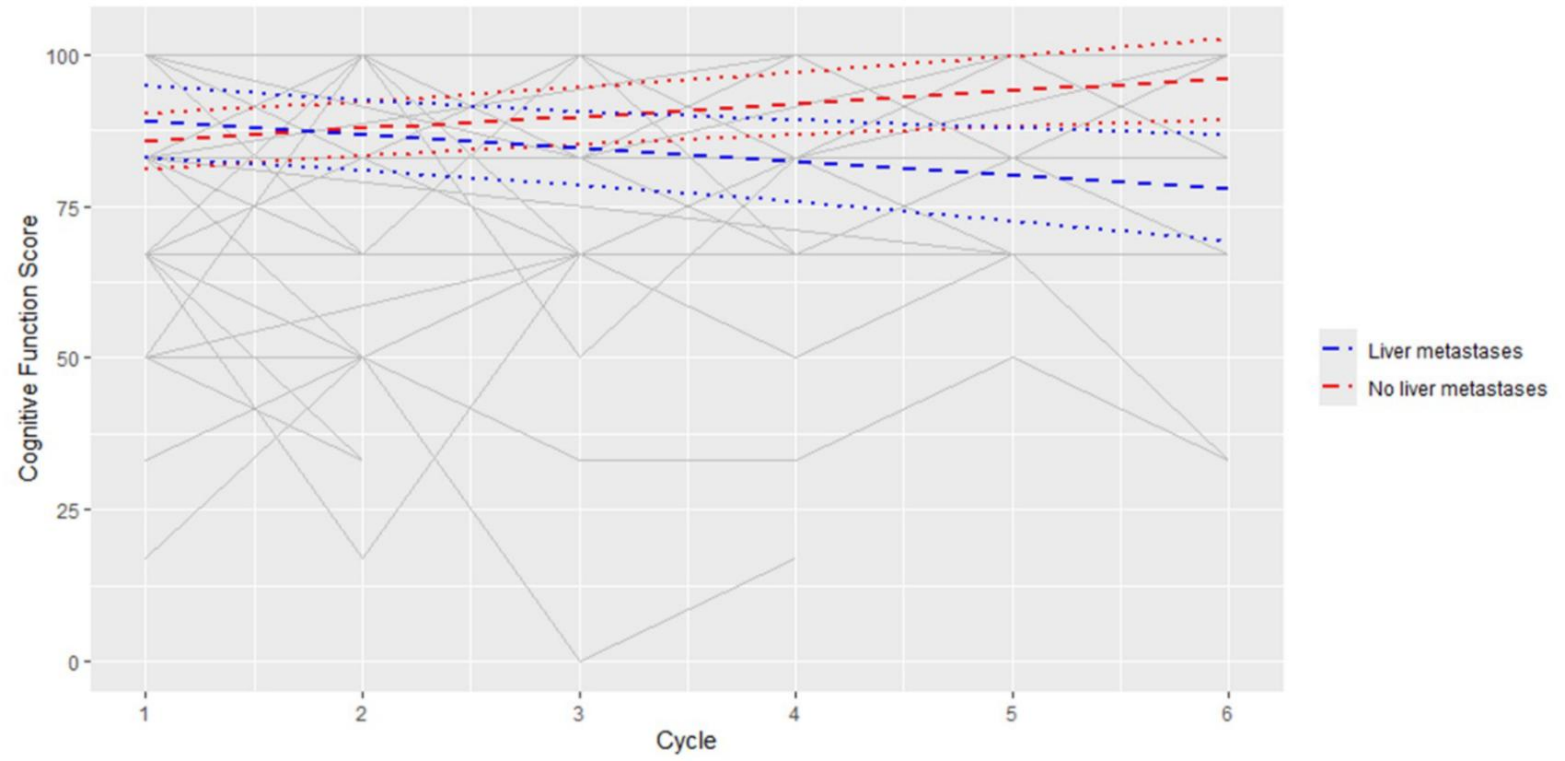
Among 122 participants, the median CFS at baseline was 84. This score is derived using the EORTC-QLQ-C30 Scoring Manual which converts the raw score out of 4 to a toxicity score out of 100. The individual grey lines are data points from the individual participants. For patients without liver metastases, there was a significant increase in CFS score of 2.1 per cycle (95% CI = 0.88 to 3.26, *P* = .001), while for patients with liver metastases, there was a significant reduction in CFS of −2.2 per cycle (95% CI = −3.76 to −0.62, *P* = .0072).

Among 122 participants, the median CFS at baseline was 84. CFS deterioration, defined by a 10-point decrease from baseline to EoT, occurred in 25/122 (20%), while CFS improvement, defined by a 10-point increase from baseline to EoT, occurred in 20/122 (16%). Changes in CFS over time are demonstrated in Figures 3 and 4. A significant reduction in CFS was observed for patients in lower ECOG groups of −1.2 per cycle (95% CI = −2.1 to −0.2, *P* = .0165) (Figure 3). At baseline, there was no significant difference in CFS between patients with and without liver metastases (Estimate = 3.25, 95% CI = −3.7 to 10.2, *P* = .3553) (Figure 4). For patients without liver metastases, there was a significant increase in CFS score of 2.1 per cycle (95% CI = 0.88 to 3.26, *P* = .001), while for patients with liver metastases, there was a significant reduction in CFS of −2.2 per cycle (95% CI = −3.76 to −0.62, *P* = .0072). This resulted in there being significant decreases in CFS between patients with and without liver metastases at Cycles 4 (Estimate = −9.5, 95% CI = −17.52 to 1.54, *P* = .0201), 5 (Estimate = −13.79, 95% CI = −22.89 to −4.69, *P* = .0035), and 6 (Estimate = −18.05, 95% CI = −28.48 to −7.63, *P* = .001).

### Emotional Function Score

**Figure 5.**
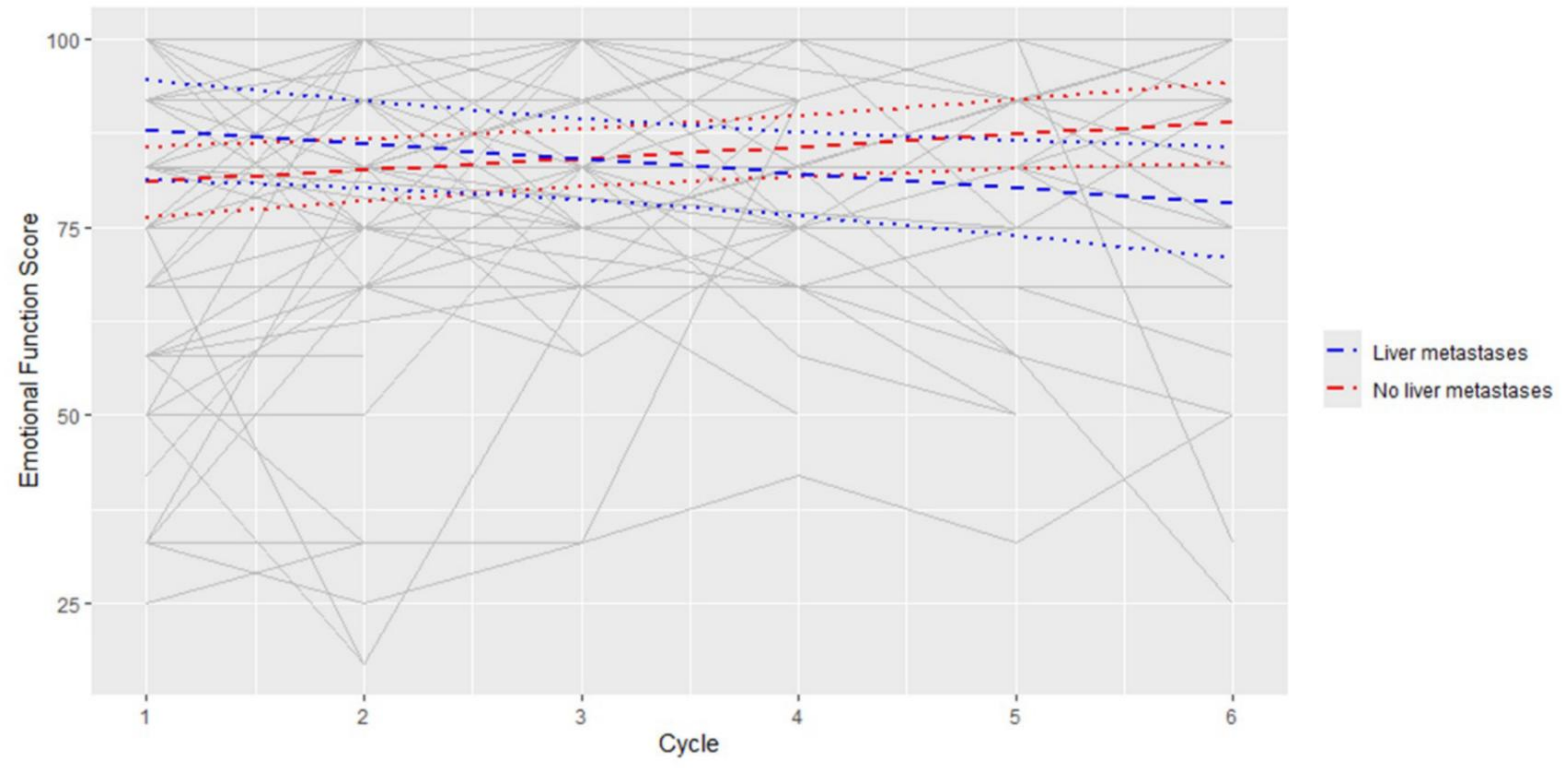
Baseline Emotional Function score for all participants pre-Cycle 1 was 77/100. This score is derived using the EORTC-QLQ-C30 Scoring Manual which converts the raw score out of 4 to a toxicity score out of 100. On average, there was an increase in the EFS for patients without liver metastases of 1.57 per cycle (95% CI = 0.26 to 2.9, *P* = .0199) and a decrease in the EFS for patients with liver metastases of −1.94 per cycle (95% CI = −3.75 to −0.14, *P* = .0355).

Among 122 participants, the median EFS at baseline was 77. EFS deterioration, defined by a 10-point decrease from baseline to EoT, occurred in 14/122 (11%), while EFS improvement, defined by a 10-point increase from baseline to EoT, occurred in 14/122 (11%). Changes in EFS over time are demonstrated in Figure 5. The final multivariable model includes cycle number, liver metastases, and an interaction between cycle number and liver metastases (*P* = .0025). There was no significant difference in EFS between patients with and without liver metastases (Estimate = 6.9, 95% CI = −1.14 to 15.01, *P* = .0913). On average, there was an increase in the EFS for patients without liver metastases of 1.57 per cycle (95% CI = 0.26 to 2.9, *P* = .0199) and a decrease in the EFS for patients with liver metastases of −1.94 per cycle (95% CI = −3.75 to −0.14, *P* = .0355). This resulted in a difference of −10.6 in the EFS for patients with liver metastases at Cycle 6 compared to patients without liver metastases (95% CI = −19.91 to −1.44, *P* = .0242). A summary of Global Health Status, Physical Function, Emotional Function, and Cognitive Function Scores is presented in Table 2.

**Table 2.** Summary of Global Health Status, Physical Function, Emotional Function, and Cognitive Function Scores.

	Global Health Status	Physical Function Score	Cognitive Function Score	Emotional Function Score
Median score at baseline	67	83	84	77
Worse at end of trial, *n* (%)	29 (24%)	30 (25%)	25 (20%)	14 (11%)
Unchanged at end of trial, *n* (%)	77 (63%)	86 (70%)	77 (64%)	94 (78%)
Better at end of trial, *n* (%)	16 (13%)	6 (5%)	20 (16%)	14 (11%)

## Discussion

The PEARLER study is the first prospective real-world data investigating HRQoL in patients undergoing EP-CTs across multiple clinical trial sites and studies. The study demonstrates that for most patients, GHS, PFS, EFS, and CFS, which are key determinants in overall HRQoL, remain stable through the EP-CT. It is known that for most patients who enroll into EP-CTs, their expectations for treatment benefit exceed those of their physicians.^9^ These trials are a source of hope for patients who have exhausted standard therapies and hence often during the informed consent process, the discussion mainly focuses on theoretical toxicity risks and therapeutic benefits.^10^ More recently, it has been demonstrated through survey-based analysis that patients who enroll into EP-CTs often have different concerns to trial participation than clinicians.^11^ There has been a greater emphasis on HRQoL and high value healthcare provision as patients exhaust standard treatment options, and hence, the focus of discussion for many EP-CT participants has become more holistic. The results through PEARLER have demonstrated that for most participants, HRQoL remains steady, and these data allow patients to make informed choices about enrolling into clinical trials that extend beyond discussions regarding unknown therapeutic benefit and systemic toxicities.

In addition to understanding the impact of EP-CTs on the participants holistically, several researchers have commented that incorporation of PROM-based HRQoL assessments can help better determine dose-limiting toxicities (DLTs).^5^^,^^11^ This is suggested from the observation that healthcare providers often underdetect symptoms or underestimate their severity, especially when those symptoms are non-life threatening but are impacting HRQoL.^12^ Hence, there is significant potential benefit to including PROM as part of EP-CT protocol design. PROM analysis in clinical trials, especially in the phase II and phase III setting, is now routine, has influenced drug registration globally, and is recommended by both European and American guidelines. Many studies use change in GHS or PFS scores over time, often also known as time-to-deterioration (TTD), to justify the utility of a therapeutic agent if standard measurements of success such as progression free survival or overall survival are not met.^13^^,^^14^ Barriers exist to using PROMs in EP-CT largely because their general utility in this space is unknown.^2^ Even in the phase II/III setting, the benefit of routine PROM collection is theoretical, while in practice, improvement in clinical outcomes directly attributable to PROM-related research is less established.^15^ Our study has established that PROM-related research across EP-CTs is feasible and can lead to clinically meaningful data collection.

PROM-based analysis of HRQoL can provide understanding of the impact of clinical trials on patients that extend beyond physical measures of health. In our study, we assessed for cognitive and emotional changes in patient outcomes during their clinical studies. Although for most participants, there was no significant improvement or deterioration in these scores, we did note that 20% had a decrease in their CFS while 11% had a decrease in their EFS. The median age of participants in many studies focusing on EP-CTs has been the mid-60s,^16^^,^^17^ and thus, the impact of trial participation on cognitive impairment is an important factor for consideration when enrolling patients. This is especially relevant as our cancer population increases in age, and data show that elderly patients do just as well on EP-CTs as their younger counterparts.^18^ Up to 50% of patients undergoing cancer therapy have some cognitive impairment directly attributable to their cancer journey.^19^ Our study also demonstrates the critical nature of disease burden and how it can play a role in quality of life. Liver metastases are known to be a poor prognostic factor in multiple cancers and some scoring modalities to predict outcomes in EP-CTs have used liver metastases as a negative predictive marker.^20^ What was new in our study, however, was the impact this disease burden can have on cognitive and emotional function. Although the presence of liver metastases is associated with increased toxin build up in the blood, which can be associated with fatigue and confusion in the setting of end stage liver failure and encephalopathy, such changes in CFS and EFS are novel findings. Thus, in the EP-CT space, prior to this study, the impact of trial participation on cognitive impairment is unknown. It should be noted that the PEARLER study does not accurately assess cognitive impairment formally but rather assesses the patient’s perspective of their cognitive functioning. Formal testing of cognitive impairment during EP-CT participation in a prospective manner has not been published but researchers are investigating this given its known issue in cancer care.^21^

Our findings can also be situated within the broader HRQoL literature in early-phase oncology. A systematic review by Fiteni et al. demonstrated that HRQoL has rarely been assessed in phase I studies, with only 1.1% of 1333 trials reporting HRQoL endpoints.^2^ More recently, Anota et al. showed that HRQoL data could inform recommended phase II dose selection, supporting the potential role of patient-reported outcomes in guiding dose-finding decisions.^4^ Henon et al. further highlighted that patients’ perceptions of tolerability often diverge from clinician-reported adverse events, underscoring the unique value of direct patient input in early-phase trials.^11^ Lai-Kwon et al. surveyed key stakeholders and noted both enthusiasm for and barriers to implementing HRQoL collection in dose-finding studies, including concerns about workload and analytic complexity.^5^ Against this backdrop, PEARLER provides the first prospective, multi-center dataset confirming that systematic HRQoL assessment in phase I/Ib trials is feasible, acceptable to patients, and capable of yielding novel insights—such as the impact of liver metastases on HRQoL trajectories—not previously described in the literature.

There are limitations to our study however, mainly due to the lack of a control group. Given this is a non-randomized cohort study across a large number of EP-CTs with different therapeutics, it is not possible to determine if these toxicities are more prominent in certain types of trials in our cohort alone. Another limitation of this study is the heterogeneity of trial designs. Participants were enrolled across multiple phase I/Ib trials with differing cycle lengths, visit schedules, mandatory procedures (such as biopsies), and investigational drug classes. These design features may independently influence HRQoL and introduce variability not fully accounted for in our analyses. It is important to note that PEARLER was not designed to individualize HRQoL outcomes per trial, but rather to provide the first prospective real-world evidence that HRQoL assessments are feasible and informative across a broad spectrum of early-phase studies. While heterogeneity in trial design, treatment regimens, and eligibility criteria could contribute to variability, we view this as reflective of real-world phase I/Ib practice. Our goal was to capture patient experience across this landscape rather than evaluate single-trial effects. Larger datasets in the future may allow stratified analyses or trial-level adjustments; however, the strength of PEARLER lies in demonstrating the feasibility and value of systematic HRQoL collection across early-phase oncology trials.

Furthermore, we cannot determine TTD due to the lack of a control arm. In order to do so, a comparative analysis between those patients who undergo EP-CTs versus those who do not would be required. However, typically participants who enroll into EP-CTs do so as a final hope for their cancer treatment. The majority of those who do not, ie, those who could be in a control arm, would not be suitable for EP-CTs due to frailty or failure during screening. These participants are likely to be more frail and therefore comparative analysis would be flawed due to heterogenous populations between the two arms. Therefore, PEARLER provides the most comprehensive analysis of HRQoL in EP-CTs to date, with future directions needing to be focused on analysis of HRQoL based on therapeutic modalities, types of studies, and patient populations of interest. Furthermore, it is well documented that participants in early-phase trials may under-report adverse events or declines in quality of life due to concerns about being withdrawn from treatment. Such reluctance could lead to underestimation of HRQoL deterioration in our study cohort. Survivorship bias is an important consideration in longitudinal HRQoL studies, as patients who remain longer on trial may differ systematically from early dropouts. In PEARLER, however, baseline performance status (ECOG) was similar between patients who completed only Cycle 1 and those who contributed multiple cycles, with only a modest enrichment of ECOG 0 among those who completed ≥4 cycles. This suggests that early attrition was not primarily driven by poorer baseline functional status and that survivorship bias is unlikely to have substantially distorted our findings.

These findings have important implications for trial design, patient counseling, and supportive care. From a trial design perspective, incorporating HRQoL assessments into early-phase studies can provide valuable complementary data to toxicity grading and pharmacokinetics, potentially refining dose-escalation and expansion decisions. For clinicians, the observation that most patients maintained stable HRQoL through participation allows for more balanced and evidence-based discussions with prospective participants, countering fears that enrolment will inevitably diminish quality of life. Finally, the identification of subgroups at risk of poorer HRQoL trajectories (eg, patients with liver metastases) highlights opportunities for targeted supportive care interventions during early-phase trial participation, such as proactive cognitive and emotional support.

## Conclusions

Overall, the PEARLER study provides the first prospective real-world evidence regarding HRQoL of participants through their EP-CT journey. Reassuringly for the majority of patients, HRQoL, when assessed using GHS, PFS, EFS, and CFS, remains stable within the limits of the analysis. Although there are participants who have a deterioration in HRQoL, clinicians should use such data to inform prospective participants who plan to enroll into EP-CTs regarding the impact such trials can have on HRQoL in addition to tumor response. Further prospective studies with larger datasets could be used to create predictive models to assess such responses and plan interventions to preserve and support HRQoL during clinical trial participation.

## Supplementary Material

pkaf108_Supplementary_Data

## Data Availability

The datasets for this manuscript are not publicly available but requests to access the datasets should be directed to Udit Nindra (udit.nindra@health.nsw.gov.au).
